# Predicting postinterventional rupture of intracranial aneurysms using arteriography-derived radiomic features after pipeline embolization

**DOI:** 10.3389/fneur.2024.1327127

**Published:** 2024-03-07

**Authors:** Chao Ma, Shikai Liang, Fei Liang, Ligong Lu, Haoyu Zhu, Xianli Lv, Xuejun Yang, Chuhan Jiang, Yupeng Zhang

**Affiliations:** ^1^School of Clinical Medicine, Tsinghua University, Beijing, China; ^2^Department of Neurosurgery, Beijing Tsinghua Changgung Hospital, School of Clinical Medicine, Tsinghua University, Beijing, China; ^3^Institute for Intelligent Healthcare, Tsinghua University, Beijing, China; ^4^Department of Vascular Surgery and Interventional Radiology, Peking University Third Hospital, Beijing, China; ^5^Guangdong Provincial Key Laboratory of Tumor Interventional Diagnosis and Treatment, Zhuhai Interventional Medical Center, Zhuhai Hospital, Affiliated with Jinan University, Zhuhai, China; ^6^Department of Neurosurgery, Beijing Tiantan Hospital, Capital Medical University, Beijing, China

**Keywords:** angiography, hemodynamics, intracranial aneurysm, perfusion, propensity score

## Abstract

**Background and purpose:**

Postinterventional rupture of intracranial aneurysms (IAs) remains a severe complication after flow diverter treatment. However, potential hemodynamic mechanisms underlying independent predictors for postinterventional rupture of IAs remain unclear. In this study, we employed arteriography-derived radiomic features to predict this complication.

**Methods:**

We included 64 patients who underwent pipeline flow diversion for intracranial aneurysms, distinguishing between 16 patients who experienced postinterventional rupture and 48 who did not. We performed propensity score matching based on clinical and morphological factors to match these patients with 48 patients with postinterventional unruptured IAs at a 1:3 ratio. Postinterventional digital subtraction angiography were used to create five arteriography-derived perfusion parameter maps and then radiomics features were obtained from each map. Informative features were selected through the least absolute shrinkage and selection operator method with five-fold cross-validation. Subsequently, radiomics scores were formulated to predict the occurrence of postinterventional IA ruptures. Prediction performance was evaluated with the training and test datasets using area under the curve (AUC) and confusion matrix-derived metrics.

**Results:**

Overall, 1,459 radiomics features were obtained, and six were selected. The resulting radiomics scores had high efficacy in distinguishing the postinterventional rupture group. The AUC and Youden index were 0.912 (95% confidence interval: 0.767–1.000) and 0.847 for the training dataset, respectively, and 0.938 (95% confidence interval, 0.806–1.000) and 0.800 for the testing dataset, respectively.

**Conclusion:**

Radiomics scores generated using arteriography-derived radiomic features effectively predicted postinterventional IA ruptures and may aid in differentiating IAs at high risk of postinterventional rupture.

## 1 Introduction

The pipeline embolization device (PED; ev3 Neurovascular, Irvine, CA, United States), a stent that diverts blood flow, has been approved for treating large or wide-necked proximal carotid aneurysms. Recently, this device has gained popularity because of its ability to promote complete occlusion (1). As many multicenter studies have shown, postinterventional hemorrhage complications, especially postinterventional rupture (PIR) of intracranial aneurysms (IAs) after flow-diverter placement, remain a concern, with a incidence of 2%–4% and high fatality (2, 3). Lately, a considerable amount of research has focused on identifying the predictors of IA PIR. Independent predictors, such as a history of subarachnoid hemorrhage, large aneurysm size, and location in the posterior circulation, have been identified (4–6). However, the potential hemodynamic mechanisms underlying these findings remain unclear. To address this gap, our study underscores the importance of identifying quantitative hemodynamic features. Such an approach is essential for developing an objective scoring model to accurately assess the risk of postoperative rupture. This methodological advancement aims to enhance predictive accuracy and support clinical decision-making.

Radiomics is a newly developed approach that allows feature extraction from various images and enables the quantitative analysis of image features (7, 8). Given that angiographic parametric imaging can generate multiple real-time flow dynamic parameters and color-encoded maps based on contrast media flow in the vasculature (9, 10), we aimed to use arteriography-derived hemodynamic radiomic features to explore the feasibility of predicting IA PIR via a radiomics approach.

## 2 Methods

### 2.1 Patient enrollment

We retrospectively screened the records of patients treated with a PED from the databases of three centers (xxxx) between June 2015 and July 2021. As the data used in this study were retrospective and de-identified, institutional review board approval was not required, and the requirement for informed consent was waived. For each patient in the PIR group, we enrolled three matching controls without PIR for the postinterventional unruptured (PIU) group. Propensity score matching was performed based on patient age, sex, aneurysm size, aneurysm location, and the number of PEDs used to balance the patient backgrounds between the PIR and PIU groups.

Clinical variables and outcomes were collected from medical records, angiographic images, and telephone questionnaires. Patients with delayed aneurysmal subarachnoid hemorrhage after PED placement were identified from computed tomography scans, and those with intraparenchymal hemorrhages were excluded. The inclusion criteria were as follows: (1) IAs with successful PED placement and (2) IAs with PIR within the early period (<3 months) post-implantation or without PIR. The exclusion criteria were as follows: (1) recurrent IAs, (2) ruptured IAs, (3) IAs treated with PED-assisted coiling, (4) IAs without angiographic imaging data, and (5) IAs without clinical and angiographic follow-up outcomes. The dataset was randomly divided into two subsets, namely the training and independent test datasets, at a 3:1 ratio.

Patients undergoing endovascular treatment received clopidogrel (75 mg/day) and aspirin (100 mg/day) orally for 7 days before the procedure. Thromboelastography was performed to examine platelet activity inhibition before surgery, with an inhibition rate of <50% indicating hyporesponsiveness to arachidonic acid and a rate of 30%–90% indicating a normal adenosine diphosphate level. All procedures were performed under general anesthesia and systemic heparinization.

### 2.2 Arteriography image preprocessing and time density curve parameter calculation

Digital subtraction angiography (DSA) data were obtained from different stations, including Artis Station (Siemens, Munich, Germany), Terra Station (GE Healthcare, Chicago, IL, United States), AW6302 Station (GE Healthcare), and 722,038-153 Station (Philips, Amsterdam, The Netherlands), at the work position. An injection pump was utilized to administer the contrast media. Each DSA run was conducted during the injection of 4 mL/s Vispaque (GE Healthcare Ireland Limited, Carrigtohill, Munster, Ireland) via a 5-F angiographic catheter into the cervical segment of the internal carotid artery, with a total volume of 6 mL. The acquisition parameters for most DSA sequences were as follows: pixel spacing, 0.154 × 0.154; median peak tube voltage, 82.1 kV (interquartile range (IQR): 76.7–85.9 kV); window center, 2047; window width, 4,095; cine rate, 4; median number of frames for each Digital Imaging and Communications in Medicine (DICOM) file, 32.0 (IQR: 21–41); and row and column size of each frame, both 1,024. Imaging data were acquired from the final DSA run immediately following the FD implantation procedure. No additional angiography was performed in the days following implementation. The imaging protocol utilized working views with rotation and magnification to optimize aneurysm visualization, ensuring no superimposition of vessels over the aneurysm. This approach was uniformly applied across all patients in the study to maintain consistency and reliability of the radiomic feature extraction.

**Figure 1:**
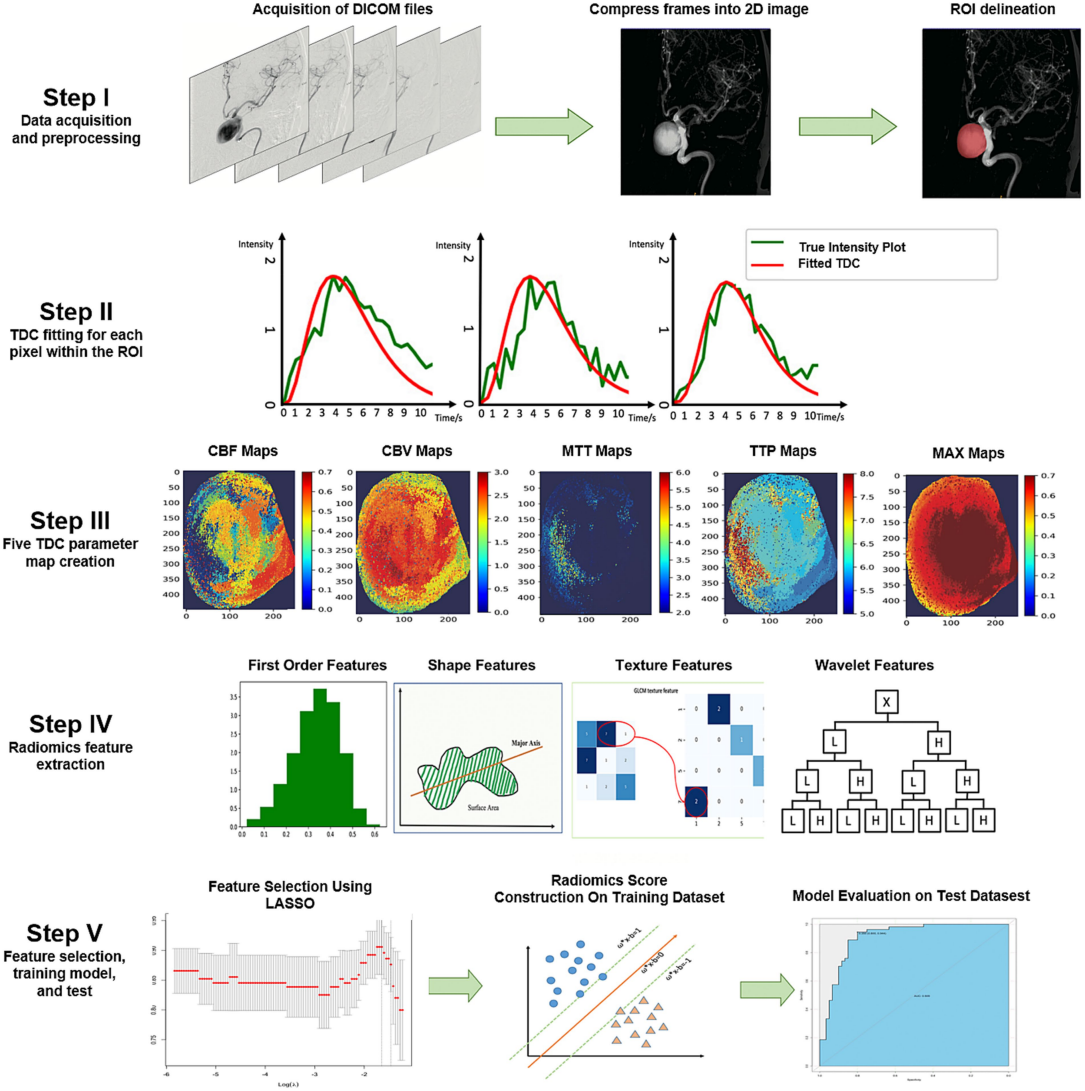
Illustration of the study process. The DICOM files were compressed into one frame, and ROIs were delineated by two neuro-interventionalists who were blinded to each other’s results (Step I). A pixel-wise calculation of the time density curve was performed (Step II), and five perfusion parameters were derived from the time density curve for each pixel. Subsequently, five perfusion maps were generated for each patient (Step III). We extracted five groups of perfusion-related radiomics features on each map, including first-order features, shape features, texture features, and wavelet features (Step IV). Informative features were selected by the LASSO, a radiomics score model was constructed to discriminate the PIR and PIU groups, and the model performance was further tested on an independent test dataset (Step V). 2D, two-dimensional; CBF, cerebral blood flow; CBV, cerebral blood volume; DICOM, Digital Imaging and Communications in Medicine; LASSO, least absolute shrinkage and selection operator; MAX, maximum contrast media concentration; MTT, mean transit time; PIR, postinterventional rupture; PIU, postinterventional unruptured; ROI, region of interest; TDC, time density curve; TTP, time to peak.

The overall procedure of this study is illustrated in Figure 1. The DICOM files were compressed into a single frame for each DSA run to display all pixels opacified by the contrast agent on a two-dimensional (2D) image (Figure 1, Step I). We then used a simplified gamma variate function to fit the time density curve (11) and codes for image preprocessing (Figure 1, Step II). The time density curve fitting program, developed in Python (version 3.6.1), builds upon computational methods previously outlined in our related publications (10). The time density curve was used to calculate the following five contrast flow-related parameters (Figure 1, Step III): cerebral blood flow, cerebral blood volume, mean transit time, time to peak, and maximum contrast media concentration (MAX). The definition of each parameter has been clarified previously (12).

### 2.3 Radiomics feature extraction

The region of interest (ROI) on the IAs was independently delineated by two interventional neuroradiologists who were blinded to group allocation. ROI delineation was meticulously performed by two experienced interventional neuroradiologists, focusing exclusively on the aneurysm using working views with rotation. This method was chosen to eliminate vessel overlap and enhance the accuracy of radiomic analysis. We used PyRadiomics (version 3.0), an open-source Python package, to extract 1,459 radiomics features from each DSA run (13). Overall, 290 radiomics features, including 18 first-order statistical features, 24 gray-level co-occurrence matrix texture features, 16 gray-level run-length matrix texture features, and 232 wavelet features, were extracted for each parameter map. We also extracted nine additional 2D shaped features from the time-to-peak map. The radiomics features were named based on the parameter maps, image types, feature classes, and feature names; for example, “TTPoriginal_shape2D_MinorAxisLength” is the feature MinorAxisLength belonging to the shape2D class, extracted on an original time-to-peak map.

### 2.4 Dimensionality reduction and radiomics score construction

Radiomics feature stability was assessed by calculating the intraclass correlation coefficient (ICC) between two ROI contours; only features with ICCs ≥0.8 were considered stable and entered into the following classifier construction. Subsequently, three steps were adopted to select the features in the training cohort. First, all selected stable features were tested using the independent samples *t*-test or Mann–Whitney *U* test to determine potentially important features. Features that did not pass either of the tests were excluded. Second, the least absolute shrinkage and selection operator (LASSO) method was employed to improve the prediction accuracy and interpretability of the statistical model. Third, we computed Spearman’s correlation coefficient for the LASSO-selected features to account for any significant linear dependencies. Highly correlated features (0.90–1.00) were considered to have severe linear dependence. Finally, the radiomics score was calculated using the following formula:


$$\mathrm{Radiomics}\;\mathrm{score}=\left(\sum \beta_{i}\times X_{i}\right)+\mathrm{intercept}\;\left(i=0,1,2,3\right)$$

where *X_i_* represents the *i*th selected feature and *β_i_* is its coefficient.

The Youden index was used to determine the optimal cutoff point for the radiomics score, aiming to best distinguish between the PIR and PIU groups. The cutoff point was established as the radiomics score that maximized Youden’s *J* statistic, defined as:


J=Sensitivity+Specificity−1

### 2.5 Prediction performance evaluation and statistics

Prediction performance of the training dataset was evaluated using the area under the curve (AUC) of the receiver operating characteristic curve. The sensitivity, specificity, positive predictive value, negative predictive value, and Youden index were also calculated. The Shapiro–Wilk test was used to test the data normality. When representing continuous variables, differences were assessed using a *t*-test or Mann–Whitney *U* test, as appropriate, and the data are represented as the median and IQR. We adopted the chi-squared test to evaluate differences in categorical variables, and the results are presented in terms of the number of events and relative frequency (%). Statistical significance was defined as *p* ≤ 0.05. Statistical analyses were performed using R software (version 3.6.3, R Foundation for Statistical Computing, Vienna, Austria). The additional R packages used in this study were “glm,” “OptimalCutpoints,” and “ggplot2.”

## 3 Results

### 3.1 Propensity score matching and baseline demographics

**Figure 2:**
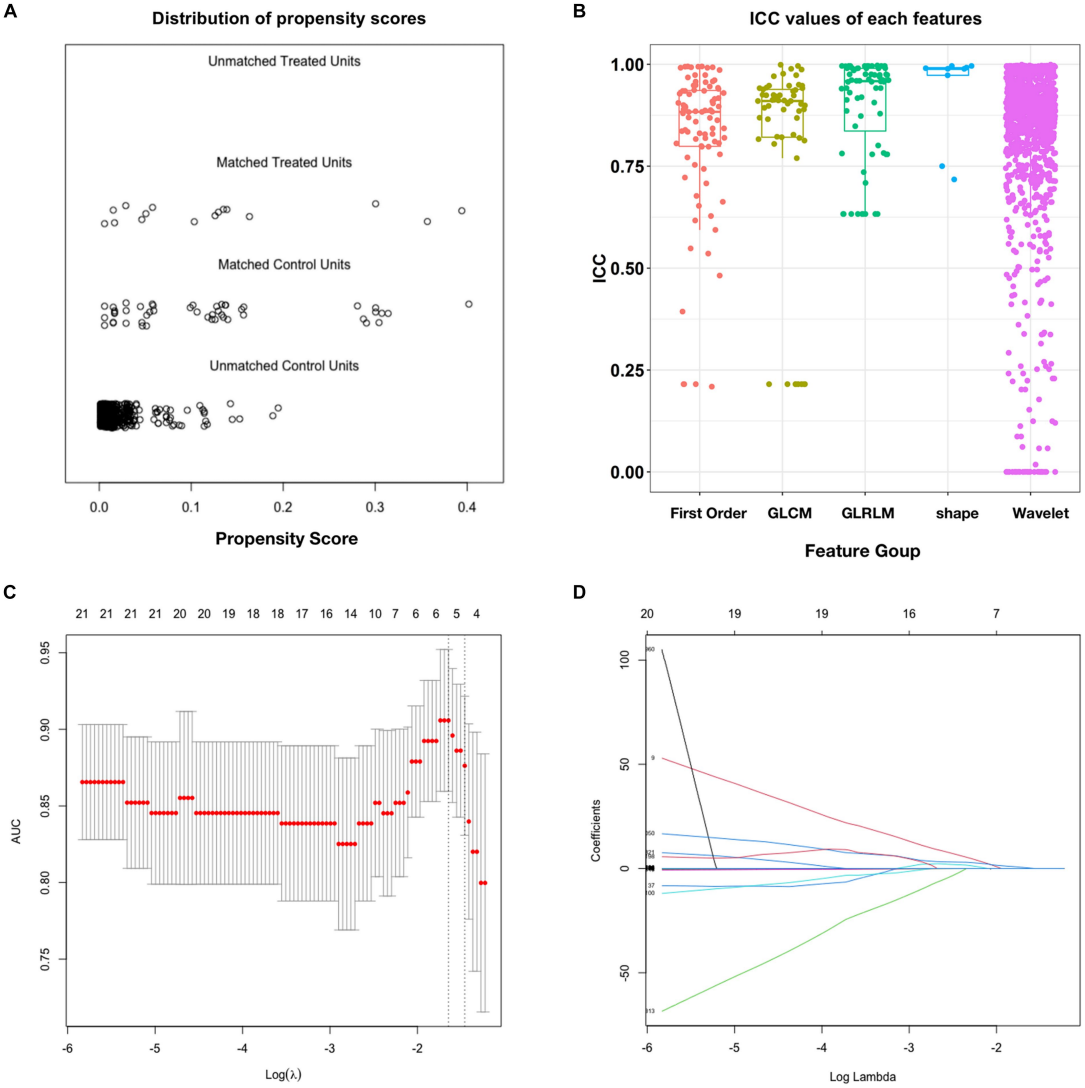
Propensity score matching and intraclass correlation coefficient results. **(A)** Distribution of propensity scores. **(B)** Boxplot of ICC values for the features extracted from the five feature groups. **(C)** Feature selection in the training dataset; this method minimizes the sum of the squares of residues, with the sum of the absolute values of the selected feature coefficients not exceeding the tuning parameter (*λ*). We used five-fold cross-validation to tune the parameter (*λ*) selection in the LASSO model. The AUC was plotted versus log (*λ*). Six features with non-zero coefficients were selected using the minimum criteria. **(D)** LASSO coefficient profiles of features in the training dataset. Each colored line represents the coefficient of each feature. AUC, area under the curve; GLCM, gray-level co-occurrence matrix; GLRLM, gray-level run-length matrix; ICC, intraclass correlation coefficient; LASSO, least absolute shrinkage and selection operator.

We selected 308 patients with unruptured IAs treated with PEDs from the database, of whom 16 patients who experienced postinterventional IA rupture were included in the PIR group. After propensity score matching, each aneurysm in the PIR group was successfully matched with three aneurysms from the remaining patients in the database (PIU group, *n* = 48). The distribution of propensity scores showed that the PIR and PIU groups were well-balanced (Figure 2A). The 64 patients from the PIR and PIU groups were randomly divided into the training and test datasets (*n* = 43 and 21, respectively) at a 2:1 ratio, with two-thirds of the 16 patients with delayed rupture assigned to the training cohort and one-third to the test cohort. This strategy, combined with five-fold cross-validation, enhances our model’s robustness and generalizability. As shown in Table 1, the baseline characteristics showed no significant differences between the two datasets.

**Table 1:** Comparison of the baseline characteristics between the PIR and PIU groups.

Characteristic	PIR group (*n* = 16)	PIU group (*n* = 48)	*p*-value	Training group (*n* = 43)	Test group (*n* = 21)	*p*-value
Age, years, median (IQR)	53.0 (43.0–56.3)	53.0 (48.0–63.0)	0.248	54.0 (50.0–63.0)	51.0 (46.5.0–56.0)	0.066
Sex, male/female	8/8	22/26	0.772	21/22	9/12	0.653
Aneurysm size, mm	21.6 ± 8.3	17.1 ± 7.5	0.245	15.6 ± 7.4	19.5 ± 7.8	1
Morphology, saccular/non-saccular	12/4	38/10	1	34/9	16/5	1
Location	—	—	0.201	—	—	0.105
ICA	12	25	—	21	16	—
MCA	2	6	—	7	1	—
VA	2	17	—	15	4	—
PED number, single/multiple	15/1	44/4	1	3/40	2/19	1

ICA, internal carotid artery; IQR, interquartile range; MCA, middle cerebral artery; PED, pipeline embolization device; PIR, postinterventional rupture; PIU, postinterventional unruptured; VA, vertebral artery.

### 3.2 Informative radiomics features and prediction score construction

After ICC analysis, 86.6% (1,264/1,459) of the radiomics features were deemed stable. Figure 2B illustrates the ICC values for each feature across all groups. The detailed percentages of the stable features in each perfusion map are summarized in Supplementary Table S1. Based on independent samples *t*-tests, 179 of the 1,264 stable features were found to be significant (*p* < 0.05), with 230 exhibiting a Gaussian distribution with homoscedasticity. The remaining 1,034 features were subjected to Mann–Whitney *U* tests, which revealed that 1,002 features were significantly different (*p* < 0.05). Consequently, these 1,181 features were selected for LASSO regression. Overall, 1,181 features passed the scrutiny of the *t*-test or Mann–Whitney *U* test. Finally, the LASSO algorithm selected six informative features with non-zero coefficients (Figures 2C,D), none of which showed high correlation.

One feature was extracted from the shape2D feature class, named “Original_shape2D_MinorAxisLength”; one feature was extracted from the cerebral blood volume angiographic parametric image, named “CBVwavelet.LL_glrlm_ShortRunLowGrayLevelEmphasis”; and four features were extracted from MAX angiographic parametric images, two of which were energy features, namely “MAXwavelet.LH_firstorder_Energy” and “MAXwavelet.HH_firstorder_Energy,” while the other two were total energy features, namely “MAXwavelet.LH_firstorder_TotalEnergy” and “MAXwavelet.HH_firstorder_TotalEnergy.”

The detailed coefficients for each feature are listed in Supplementary Table S2. The six selected features were used to construct the radiomics score by multiplying the value of each feature by its coefficient and then summing all six products with the intercept.

### 3.3 Model performance evaluation and feature ranking

**Figure 3:**
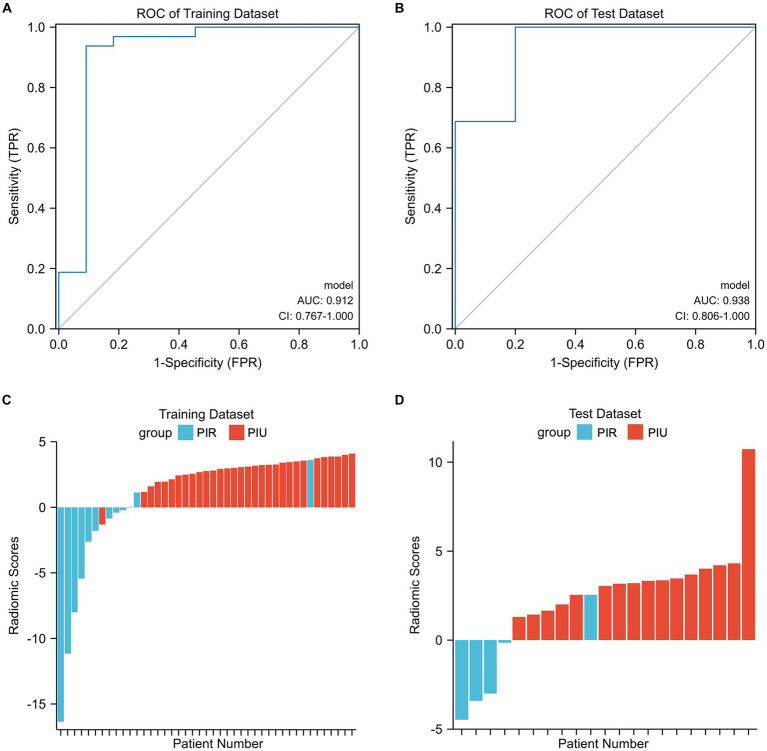
**(A,B)** Illustration of the five-fold cross-validated ROC curve of the radiomics score model on the training and test datasets. **(C,D)** The bar plots for the training and test datasets with the prediction value for each patient. AUC, area under the curve; CI, confidence interval; PIR, postinterventional rupture; PIU, postinterventional unruptured; ROC, receiver operating characteristic.

In the training dataset, the radiomics score ranged from −16.347 to 4.091, and the mean radiomics scores in the PIR and PIU groups were −3.829 and 2.764, respectively. In the test dataset, the radiomics score ranged from −4.469 to 10.722, and the mean radiomics scores in the PIR and PIU groups were −3.330 and 1.695, respectively. The optimal cutoff point of the radiomics score for differentiating between the two groups was 1.143; with this cutoff point, the radiomics score could discriminate PIR with an AUC of 0.912 (95% confidence interval: 0.767–1.000) and 0.938 (95% confidence interval: 0.806–1.000) in the training and test datasets, respectively (Figures 3A,B). The prediction results for each patient in the training and test datasets are shown in Figures 3C,D. The confusion matrix-derived score performance metrics and AUCs for the training and test datasets are presented in Supplementary Table S3.

## 4 Discussion

Postinterventional delayed aneurysm rupture is a serious complication of PED therapy. Although numerous studies have discussed possible contributing factors in terms of histopathology (14), aneurysm morphology (15), hemodynamics (6, 16, 17), and mechanical stretching after stenting (18), the underlying mechanism remains controversial. Additionally, most of these studies were case rePIRts and qualitative analyses; the results of which are limited in their generalization. Hence, in this study, we used the arteriography-derived hemodynamic radiomic features extracted from postprocedural DSA to quantitatively predict the risk of delayed rupture after PED treatment.

Previous computational fluid dynamics research has been devoted to establishing the hemodynamic factors associated with the PIR of aneurysms after flow diversion. In the earliest studies using computational fluid dynamics to analyze delayed rupture of IAs and examine the hemodynamic changes, Cebral et al. (16) used patient-specific hemodynamic analysis of three post-treatment cases in which rupture occurred and four cases in which treatment was successful. As a result, they identified a reduction in flow velocity and wall shear stress within the aneurysms and assumed that flow diversion in the parent artery increases intra-aneurysmal pressure, which may cause aneurysm rupture. Chen et al. (17) also examined hemodynamic parameters, such as streamline, blood flow velocity, aneurysm pressure, and wall shear stress, at peak systole before and after stent deployment. However, their data indicated that the velocity of blood flow entering the aneurysm did not decrease substantially. A high wall shear stress and an increase in pressure may also cause delayed aneurysm rupture.

Moreover, Li et al. (6) compared pre-and post-treatment hemodynamic changes between the delayed rupture and unruptured groups and proposed that a stable flow pattern and higher energy loss after PED placement for IAs may be important hemodynamic risk factors for delayed aneurysm rupture. Nonetheless, several widely known limitations should be considered when evaluating the results of these computational fluid dynamics analyses; these include rigid wall assumptions, physiological flow-boundary conditions that are not patient-specific, and Newtonian blood properties (19, 20). Rigid wall computational fluid dynamics models tend to overestimate pressure gradients, resulting in greater pressure increases than are actually present (21). Furthermore, the precise geometry of the stent in its deployed state is unclear, and the accuracy of the virtual stent placement technique must be improved (22).

In contrast to computational fluid dynamics analysis, which often uses many hypothetical parameters, angiographic parametric imaging is a more specific method that adds time parameters by analyzing the contrast bolus and deriving perfusion features from a time density curve. In this study, five angiographic parametric images from each individual were generated from the actual perfusion parameters of the patient. Both DSA and magnetic resonance perfusion images can generate perfusion features. Although this approach has been used in clinical settings to predict cerebrovascular malformation rupture and embolization outcomes, it has only recently been applied to IAs (10, 23, 24). These analyses highlight the potential for angiographic parametric imaging to play an important role in the clinical determination of cerebrovascular disease. Herein, we used a radiomics approach to decode the flow patterns within an ROI that included the aneurysm sac as a whole to solve the ROI placement problem and take advantage of the pattern change information hidden in pixel-wise calculated perfusion maps.

**Figure 4:**
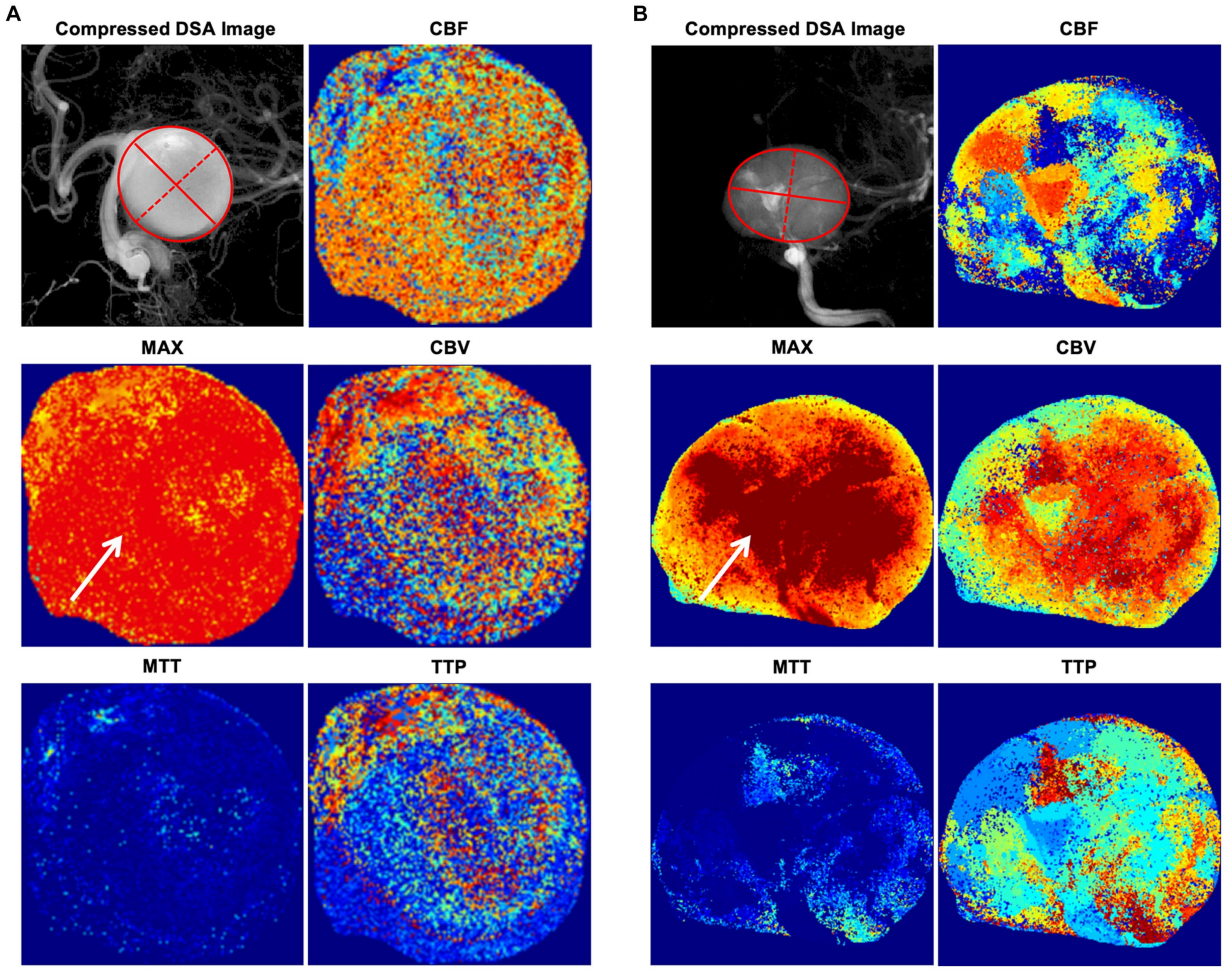
Illustrative cases of angiographic parametric imaging. **(A)** An illustrative case in the PIU group. The solid red lines represent the major axis length, and the red dotted lines represent the minor axis length. **(B)** An illustrative case in the PIR group. The solid red lines represent the major axis length, and the red dotted lines represent the minor axis length. The degree of MAX (arrows) significantly differs in the MAX angiographic parametric image between the PIR and PIU groups. CBF, cerebral blood flow; CBV, cerebral blood volume; DSA, digital subtraction angiography; MAX, maximum contrast media concentration; MTT, mean transit time; PIR, postinterventional rupture; PIU, postinterventional unruptured; TTP, time to peak.

The intra-aneurysmal pressure mechanism was also reflected in the current study via the radiomics score, which incorporated four energy-related features that were crucial in our model: MAXwavelet.LH_firstorder_Energy, MAXwavelet.HH_firstorder_Energy, MAXwavelet.LH_firstorder_TotalEnergy, and MAXwavelet.HH_firstorder_TotalEnergy. These energy and total energy features are consistent with the intra-aneurysmal pressure increase. In the traditional definition of radiomics features, the energy feature is a measure of the magnitude of voxel values in an image, and the total energy is the value of the energy feature scaled by the volume of the voxel in cubic millimeters (25). Therefore, the energy and total energy features in a MAX angiographic parametric image can be interpreted as the degree of MAX, which, then, reflects the contrast agent retention in the aneurysm to some extent. As shown in Figures 4A,B, the MAX degree significantly differed in the MAX angiographic parametric images between the PIR and PIU groups. The relationship between contrast retention and radiomic features, particularly the magnitude of voxel values, suggests a possible link to the hemodynamic behavior within the aneurysm sac. We hypothesize that decelerated inflow might indeed lead to a more magnitude of voxel values, reflecting more intense contrast retention. While we postulate that some radiomic features may reflect intrasaccular pressure, we acknowledge that this remains an assumption not yet empirically validated. Further research is needed to explore the potential connections between these radiomic features and intrasaccular hemodynamics.

The 2D shape feature, minor axis length, in our radiomics score is an indicator that can quantify the size of an aneurysm; it yields the second-largest axis length of the ROI-enclosing ellipsoid when compared to the major axis length (25). As illustrated in Figures 4A,B, most aneurysms are ellipsoid-shaped (i.e., the larger the minor axis length, the larger the major axis length and the larger the 2D area of the entire ellipse). Thus, larger minor axis lengths indicate larger aneurysm sizes. Additionally, there is evidence that larger aneurysms have a higher risk of PIR, although how aneurysm size influences intra-aneurysmal pressure has not been fully elucidated (16, 26). For saccular aneurysms of the internal carotid artery, a high aspect ratio (>1.6) is associated with aneurysm rupture (15, 27). However, we only included the minor axis length shape feature, which alone does not reflect aneurysm morphology. If we included the major axis length in our score, it would have reflected the aspect ratio of the aneurysm. Though, because of the limited sample size of this study, adding other morphological features did not improve the accuracy of the radiomics score.

Variability in DSA acquisition parameters, such as catheter position, injection rate, and mix ratio, can significantly influence the quality of imaging and subsequently affect the extraction and interpretation of radiomic features (28). As we described in method, we standardized the acquisition parameters for most DSA runs, however, we employed a multi-center approach, incorporating imaging data obtained from different machines and vendors. The scanner variability can introduce challenges in radiomics analysis. These parameters are critical in ensuring consistent and reliable imaging outcomes, which are foundational for radiomic analyses that rely on quantitative imaging features for diagnostic, prognostic, and therapeutic purposes (29, 30). Future research should aim to develop standardized DSA protocols and use advanced computational methods to reduce variability, enhancing radiomics’ reliability in clinical practice.

This study has some limitations that warrant further discussion. First, the morphology of the aneurysm itself was an important factor in the resulting PIR, and the shortcoming of the angiographic parametric imaging strategy lied in its 2D nature. Even though the working position best delineated the largest projected area of an aneurysm, the three-dimensional shape of the aneurysm was more in line with the actual situation. Second, the mechanism of aneurysm rupture following flow diversion was likely multifactorial and may include factors such as antiplatelet regimens, thrombus formation, inflammation, and the condition of the aneurysm wall. Finally, despite representing the largest cohort to date in this research context, our study’s sample size is still relatively small, highlighting the need for more extensive future studies. These efforts will aim to enhance the robustness and generalizability of our findings, further enriching our understanding and clinical application.

## 5 Conclusion

In the present study, we generated a radiomics score based on postprocedural DSA perfusion radiomics features, which predicted the PIR of IAs with acceptable accuracy after placement of the PED. This approach not only enhances our understanding of the radiomic features associated with delayed rupture, enabling clinicians to better assess rupture risk post-FD implantation but also holds significant implications for patient management and future research. Although further validation at more institutes is necessary before its widespread clinical application, we advocate for future studies to explore and integrate radiomic analysis into clinical decision-making processes.

## Data availability statement

The original contributions presented in the study are included in the article/Supplementary material, further inquiries can be directed to the corresponding authors.

## Ethics statement

Ethical approval was not required for the study involving humans in accordance with the local legislation and institutional requirements. Written informed consent to participate in this study was not required from the participants or the participants’ legal guardians/next of kin in accordance with the national legislation and the institutional requirements.

## Author contributions

CM: Conceptualization, Data curation, Formal analysis, Investigation, Methodology, Project administration, Software, Supervision, Validation, Visualization, Writing – original draft, Writing – review & editing. SL: Investigation, Methodology, Project administration, Resources, Supervision, Validation, Writing – original draft. FL: Conceptualization, Data curation, Methodology, Validation, Writing – review & editing. LL: Data curation, Formal analysis, Funding acquisition, Project administration, Resources, Supervision, Writing – review & editing. HZ: Data curation, Investigation, Methodology, Validation, Writing – original draft. XL: Data curation, Formal analysis, Investigation, Methodology, Supervision, Validation, Writing – review & editing. XY: Funding acquisition, Investigation, Project administration, Resources, Supervision, Writing – review & editing. CJ: Conceptualization, Funding acquisition, Methodology, Project administration, Resources, Supervision, Validation, Writing – review & editing. YZ: Conceptualization, Data curation, Formal analysis, Investigation, Methodology, Project administration, Software, Visualization, Writing – original draft, Writing – review & editing.

## References

[1] Kallmes DF Hanel R Lopes D Boccardi E Bonafe A Cekirge S. International retrospective study of the pipeline embolization device: a multicenter aneurysm treatment study. AJNR Am J Neuroradiol. (2015) 36:108–15. doi: 10.3174/ajnr.A4111 25355814 PMC7965920

[2] Kang H Zhou Y Luo B Lv N Zhang H Li T. Pipeline embolization device for intracranial aneurysms in a large Chinese cohort: complication risk factor analysis. Neurotherapeutics. (2021) 18:1198–206. doi: 10.1007/s13311-020-00990-8, PMID: 33447904 PMC8423892

[3] Brinjikji W Murad MH Lanzino G Cloft HJ Kallmes DF. Endovascular treatment of intracranial aneurysms with flow diverters: a meta-analysis. Stroke. (2013) 44:442–7. doi: 10.1161/STROKEAHA.112.678151 23321438

[4] Sweid A Starke RM Herial N Chalouhi N Das S Baldassari MP. Predictors of complications, functional outcome, and morbidity in a large cohort treated with flow diversion. Neurosurgery. (2020) 87:730–43. doi: 10.1093/neuros/nyz508, PMID: 31858148

[5] Rouchaud A Brinjikji W Lanzino G Cloft HJ Kadirvel R Kallmes DF. Delayed hemorrhagic complications after flow diversion for intracranial aneurysms: a literature overview. Neuroradiology. (2016) 58:171–7. doi: 10.1007/s00234-015-1615-4, PMID: 26553302 PMC4849277

[6] Li W Tian Z Zhu W Zhang YS Wang K Zhang Y. Hemodynamic analysis of postoperative rupture of unruptured intracranial aneurysms after placement of flow-diverting stents: a matched case-control study. AJNR Am J Neuroradiol. (2019) 40:1916–23. doi: 10.3174/ajnr.A6256, PMID: 31624118 PMC6975104

[7] Gillies RJ Kinahan PE Hricak H. Radiomics: images are more than pictures, they are data. Radiology. (2016) 278:563–77. doi: 10.1148/radiol.2015151169, PMID: 26579733 PMC4734157

[8] Lambin P Rios-Velazquez E Leijenaar R Carvalho S van Stiphout RG Granton P. Radiomics: extracting more information from medical images using advanced feature analysis. Eur J Cancer. (2012) 48:441–6. doi: 10.1016/j.ejca.2011.11.036, PMID: 22257792 PMC4533986

[9] Gilat Schmidt T Chen G-H Bosmans H Shiraz Bhurwani MM Ionita CN Rudin S. Initial study of the radiomics of intracranial aneurysms using angiographic parametric imaging (API) to evaluate contrast flow changes. Proc SPIE. (2019) 10948:1094805. doi: 10.1117/12.2512457

[10] Liang F Ma C Zhu H Liu L Liang S Jiang P. Using angiographic parametric imaging-derived radiomics features to predict complications and embolization outcomes of intracranial aneurysms treated by pipeline embolization devices. J Neurointerv Surg. (2021) 14:826–31. doi: 10.1136/neurintsurg-2021-017832, PMID: 34413243

[11] Chan AA Nelson SJ. (2004). Simplified gamma-variate fitting of perfusion curves. 2004 2nd IEEE International Symposium on Biomedical Imaging: Nano to Macro (IEEE Cat No. 04EX821)

[12] Zhang Y Ma C Li C Li X Liu R Liu M. Prediction of the trans-stenotic pressure gradient with arteriography-derived hemodynamic features in patients with idiopathic intracranial hypertension. J Cereb Blood Flow Metab. (2022) 42:1524–33. doi: 10.1177/0271678X221086408, PMID: 35255760 PMC9274861

[13] van Griethuysen JJM Fedorov A Parmar C Hosny A Aucoin N Narayan V. Computational Radiomics system to decode the radiographic phenotype. Cancer Res. (2017) 77:e104–7. doi: 10.1158/0008-5472.CAN-17-0339, PMID: 29092951 PMC5672828

[14] Ikeda H Ishii A Kikuchi T Ando M Chihara H Arai D. Delayed aneurysm rupture due to residual blood flow at the inflow zone of the intracranial paraclinoid internal carotid aneurysm treated with the pipeline embolization device: histopathological investigation. Interv Neuroradiol. (2015) 21:674–83. doi: 10.1177/1591019915609121, PMID: 26500232 PMC4757363

[15] Kulcsar Z Houdart E Bonafe A Parker G Millar J Goddard AJ. Intra-aneurysmal thrombosis as a possible cause of delayed aneurysm rupture after flow-diversion treatment. AJNR Am J Neuroradiol. (2011) 32:20–5. doi: 10.3174/ajnr.A2370, PMID: 21071538 PMC7964960

[16] Cebral JR Mut F Raschi M Scrivano E Ceratto R Lylyk P. Aneurysm rupture following treatment with flow-diverting stents: computational hemodynamics analysis of treatment. AJNR Am J Neuroradiol. (2011) 32:27–33. doi: 10.3174/ajnr.A2398, PMID: 21071533 PMC7964947

[17] Chen S Bai B Lv N Cheng Y Ji B. Hemodynamic analysis and implantation strategies of delayed intracranial aneurysm rupture after flow diverter treatment. Ann Transl Med. (2021) 9:1735. doi: 10.21037/atm-21-5939, PMID: 35071429 PMC8743709

[18] Fox B Humphries WE Doss VT Hoit D Elijovich L Arthur AS. Rupture of giant vertebrobasilar aneurysm following flow diversion: mechanical stretch as a potential mechanism for early aneurysm rupture. BMJ Case Rep. (2014) 2014:bcr2014011325. doi: 10.1136/bcr-2014-011325, PMID: 25355741 PMC4216898

[19] Perktold K Thurner E Kenner T. Flow and stress characteristics in rigid walled and compliant carotid artery bifurcation models. Med Biol Eng Comput. (1994) 32:19–26. doi: 10.1007/BF02512474, PMID: 8182957

[20] Benard N Perrault R Coisne D. Computational approach to estimating the effects of blood properties on changes in intra-stent flow. Ann Biomed Eng. (2006) 34:1259–71. doi: 10.1007/s10439-006-9123-7, PMID: 16799830

[21] Jodko D Jeckowski M Tyfa Z. Fluid structure interaction versus rigid-wall approach in the study of the symptomatic stenosed carotid artery: importance of wall compliance and resilience of loose connective tissue. Int J Numer Method Biomed Eng. (2022) 38:e3630. doi: 10.1002/cnm.3630, PMID: 35593678 PMC9542585

[22] Willaert WI Aggarwal R Van Herzeele I Plessers M Stroobant N Nestel D. Role of patient-specific virtual reality rehearsal in carotid artery stenting. Br J Surg. (2012) 99:1304–13. doi: 10.1002/bjs.8858, PMID: 22864891

[23] Shiraz Bhurwani MM Waqas M Podgorsak AR Williams KA Davies JM Snyder K. Feasibility study for use of angiographic parametric imaging and deep neural networks for intracranial aneurysm occlusion prediction. J Neurointerv Surg. (2020) 12:714–9. doi: 10.1136/neurintsurg-2019-015544, PMID: 31822594

[24] Zhu H Zhang Y Li C Ma C Liang F Liang S. Quantitative evaluation of the hemodynamic differences between ruptured and unruptured cerebral arteriovenous malformations using angiographic parametric imaging-derived radiomics features. Neuroradiology. (2023) 65:185–94. doi: 10.1007/s00234-022-03030-8, PMID: 35922586

[25] Zwanenburg A Vallières M Abdalah MA HJWL A Andrearczyk V Apte A. The image biomarker standardization initiative: standardized quantitative radiomics for high-throughput image-based phenotyping. Radiology. (2020) 295:328–38. doi: 10.1148/radiol.2020191145 32154773 PMC7193906

[26] Siddiqui AH Kan P Abla AA Hopkins LN Levy EI. Complications after treatment with pipeline embolization for giant distal intracranial aneurysms with or without coil embolization. Neurosurgery. (2012) 71:E509–13. doi: 10.1227/NEU.0b013e318258e1f8 22710418

[27] Ujiie H Tamano Y Sasaki K Hori T. Is the aspect ratio a reliable index for predicting the rupture of a saccular aneurysm? Neurosurgery. (2001) 48:495–503. doi: 10.1097/00006123-200103000-00007, PMID: 11270538

[28] Gao Z Zeng Y Sun J Yang J Zhou Y Zhou M. Application of low injection rate and low contrast agent dose in three-dimensional rotational digital subtraction angiography of the intracranial aneurysm. Interv Neuroradiol. (2016) 22:287–92. doi: 10.1177/1591019916631980, PMID: 26916657 PMC4984369

[29] Cobo M Menendez Fernandez-Miranda P Bastarrika G Lloret IL. Enhancing radiomics and deep learning systems through the standardization of medical imaging workflows. Sci Data. (2023) 10:732. doi: 10.1038/s41597-023-02641-x, PMID: 37865635 PMC10590396

[30] Cui Y Yin FF. Impact of image quality on radiomics applications. Phys Med Biol. (2022) 67:15TR03. doi: 10.1088/1361-6560/ac7fd7, PMID: 35803254

