# Supplementary material for: Predicting postinterventional rupture of intracranial aneurysms using arteriography-derived radiomic features after pipeline embolization
Source: Front Neurol. 2024 Mar 7;15:1327127. doi: 10.3389/fneur.2024.1327127 (PMC10954779; doi:10.3389/fneur.2024.1327127)
Supplement: Supplementary file 1 [file Table_1.DOCX]

Supplementary Material

# Supplementary Tables

**Supplementary Table 1.** Intraclass correlation coefficients of radiomics features extracted from different perfusion maps.

| **Map** | **First order** | **GLCM** | **GLRLM** | **Shape** | **Wavelet** |
| --- | --- | --- | --- | --- | --- |
| CBF | 15/18 | 21/24 | 13/16 | - | 201/232 |
| CBV | 15/18 | 21/24 | 13/16 | - | 194/232 |
| MAX | 15/18 | 21/24 | 13/16 | - | 165/232 |
| MTT | 15/18 | 24/24 | 16/16 | - | 216/232 |
| TTP | 16/18 | 24/24 | 16/16 | 9/9 | 221/232 |
| Sum | 76/90 (84.4%) | 111/120 (92.5%) | 71/80 (88.75%) | 9/9 (100.0%) | 997/1,160 (85.9%) |

CBF, cerebral blood flow; CBV, cerebral blood volume; GLCM, gray-level co-occurrence matrix; GLRLM, gray-level run length matrix; MAX, maximum contrast media concentration; MTT, mean transit time; TTP, time to peak.

**Supplementary Table 2.** Selected features in the radiomics score.

| **Radiomics feature** | **Coefficient** |
| --- | --- |
| Original_shape2D_MinorAxisLength | –0.913 |
| CBVwavelet.LL_glrlm_ShortRunLowGrayLevelEmphasis | –1.204 |
| MAXwavelet.LH_firstorder_Energy | 3.306 |
| MAXwavelet.LH_firstorder_TotalEnergy | –6.7842 |
| MAXwavelet.HH_firstorder_Energy | –0.913 |
| MAXwavelet.HH_firstorder_TotalEnergy | –1.204 |

The formula to calculate the radiomics score is as follows:

Radiomics score = –0.9133 × Original_shape2D_MinorAxisLength

– 1.2042 × CBVwavelet.LL_glrlm_ShortRunLowGrayLevelEmphasis

+ 3.3056 × MAXwavelet.LH_ first-order _Energy

– 6.7842 × MAXwavelet.LH_firstorder_TotalEnergy

– 0.9133 × MAXwavelet.HH_firstorder_Energy

– 1.2042 × MAXwavelet.HH_firstorder_TotalEnergy + 4.4827

**Supplementary Table 3.** Model performance metrics.

|  | **Training dataset** | **Test dataset** |
| --- | --- | --- |
| Sensitivity | 0.938 | 0.800 |
| Specificity | 0.909 | 1.000 |
| Positive predictive value | 0.968 | 1.000 |
| Negative predictive value | 0.833 | 0.941 |
| Youden index | 0.847 | 0.800 |
| AUC (95% CI) | 0.912 (0.767–1.000) | 0.938 (0.806–1.000) |

AUC, area under the curve: CI, confidence interval.
